# Associations Between Pathways Into Care and Service Use and Involuntary Hospitalisation Among Children and Young People

**DOI:** 10.1192/bjo.2024.268

**Published:** 2024-08-01

**Authors:** Susan Walker, Daniela Fonseca Freitas, Johnny Downs, Patrick Nyikavaranda, Sonia Johnson

**Affiliations:** 1 Division of Psychiatry, University College London, London, United Kingdom; 2 Institute of Psychiatry, Psychology and Neuroscience, King's College London, London, United Kingdom; 3 Department of Psychiatry, University of Oxford, Oxford, United Kingdom; 4 Department of Primary Care & Public Health, Brighton & Sussex Medical School, University of Sussex, Brighton, United Kingdom

## Abstract

**Aims:**

There is evidence that children and young people (CYP) of Black ethnicity are more likely to experience involuntary hospital admission. This is not explained by clinical and sociodemographic factors. One possible explanation are differences in pathways into, and/or use of mental health services (MHS). This study investigates the associations between ethnicity, pathways into MHS, MHS use and involuntary hospitalisation in CYP.

**Methods:**

Using data from the Clinical Record Interactive Search (CRIS) system for (South London and the Maudsley) SLaM services we identified 652 CYP under 18 years admitted to inpatient units between 2008 and 2021 living within the SLaM catchment; 458 (70.2%) were admitted informally and 194 (29.7%) were detained. We conducted univariable logistic regression to investigate the association between pathways into MHS (referral source, S.136 presentation), MHS use (time known to services, recent appointment prior to admission, and presence of a care plan), clinical factors (diagnosis, severity, risk) and social factors (gender, age, ethnicity, deprivation) with the outcome i.e. involuntary admission. We then conducted multivariable logistic regression to investigate the association between the clinical and social factors and involuntary admission.

**Results:**

In multivariable analyses we found evidence that adverse pathways into MHS such as S.136 presentation (OR 6.25, 95%CI 2.06-19.01, p = 0.001), and referrals from social services (OR 4.92, 95%CI 1.49-16.19, p = 0.009) and police/legal services (OR 4.22, 95%CI 1.03-17.31, p = 0.045) were associated with involuntary hospitalisation. There was no evidence that the duration of contact with MHS, having had an appointment in the 28 days prior to admission or a care plan in the 12 months prior to admission were associated with involuntary hospitalisation after adjusting for other factors. There was evidence that being of Black ethnicity (OR 2.04, 95%CI 1.19-3.50, p = 0.010), older age (13–15 years: OR 4.46, 95%CI 1.57-12.72, p = 0.005; age 16–17 years: OR 8.67, 95%CI 3.08-24.41, p < 0.001) and having a diagnosis of a psychotic disorder (OR 4.21, 95%CI 2.21-8.02, p < 0.001) were associated with involuntary admission after accounting for pathways into and use of MHS.

**Conclusion:**

In this cohort of child and adolescent inpatients living in South East London, we found that CYP who experience adverse pathways into MHS are more likely to experience involuntary hospitalisation. Prior contact with MHS did not appear to influence involuntary admission. We found that Black CYP remained more than twice as likely to be admitted involuntarily after accounting for MHS use and pathways into MHS as well as social and clinical factors.

